# The “technoscientization” of medicine and its limits: technoscientific identities, biosocialities, and rare disease patient organizations

**DOI:** 10.1007/s10202-011-0100-3

**Published:** 2011-11-10

**Authors:** Peter Wehling

**Affiliations:** University of Augsburg, Augsburg, Germany

## Abstract

The fact that the emergence of “technoscience,” resulting from the coalescing of science and technology, may have serious social and cultural impact has been debated in recent years particularly with regard to the field of medicine. The present article is exploring the scope and limits of the “technoscientization” of medicine using the example of rare disease patient associations. It is investigated whether and to what extent these organizations adopt technoscientific illness identities and subscribe to the research priorities and objectives of biomedicine. In addition, it is analyzed whether Paul Rabinow’s highly influential concept of biosociality entails a technoscientific model of identity or, quite to the contrary, offers a framework for contesting biomedical ascriptions of identities. As the article shows, patient associations do refer to technoscientific definitions of diseases yet constantly modify and transform them based on their everyday illness experiences. Likewise, the “biosociality” of rare disease patients emerges from the shared experience of having been neglected by mainstream medical research rather than from supposedly objective biomedical classifications.

## Résumé

Le fait que l’émergence de la ‘technoscience’ résultant de la coalescence de la science et de la technologie puisse avoir un impact social et culturel important a fait l’objet d’un débat important dans les dernières années en particulier dans le domaine de la médecine. Le présent article explore l’étendue et les limites de la ‘technoscientisation’ de la médecine en prenant l’exemple d’associations de patient atteints de maladies rares. L’article investigue si et dans quelle mesure ces associations adoptent des identités de maladies technoscientifiques et souscrivent aux priorités de recherche et aux objectifs de la biomédecine. De plus, l’article analyse si le concept hautement influent de biosocialité de Paul Rabinow comporte un modèle technoscientifique d’identité ou si, au contraire, il offre un cadre pour contester les attributions biomédicales des identités. Comme l’article le montre, les associations de patient réfèrent effectivement à des définitions technoscientifiques des maladies, cependant elles les modifient et les transforment constamment sur la base de leur expérience quotidienne des maladies. De même, la ‘biosocialité’ des patients atteints de maladies rares émerge de l’expérience partagée d’avoir été délaissé par le courant dominant de la recherche médicale plutôt que de classifications biomédicales supposément objectives.

## Abstract (de)

Dass die zunehmende Verschmelzung von Wissenschaft und Technik zur „Technowissenschaft“erhebliche soziale und kulturelle Auswirkungen haben kann, wird seit einigen Jahren vor allem mit Blick auf die Medizin lebhaft diskutiert. Der Beitrag untersucht die Reichweite sowie die Grenzen einer technowissenschaftlichen Transformation („technoscientization“) der Medizin am Beispiel von Patientenvereinigungen im Bereich der Seltenen Erkrankungen. Gefragt wird insbesondere danach, inwieweit diese Organisationen technowissenschaftliche Krankheitsidentitäten übernehmen und sich die Forschungsprioritäten und -ziele der Biomedizin zu eigen machen. Analysiert wird außerdem, ob der in den Sozialwissenschaften sehr einflussreiche, von Paul Rabinow geprägte Begriff der Biosozialität ein technowissenschaftliches Identitätsmodell enthält oder, im Gegenteil, einen Ansatzpunkt zur Überwindung biomedizinischer Identitätszuschreibungen bietet. Der Artikel verdeutlicht, dass die Patientenorganisationen technowissenschaftliche Krankheitsdefinitionen zwar aufgreifen, diese aber immer wieder durch lebensweltliche Erfahrungen modifizieren und transformieren. Ebenso gründet sich die „Biosozialität“von Patienten mit Seltenen Erkrankungen eher auf die geteilte Erfahrung langjähriger Vernachlässigung durch die etablierte medizinische Forschung als auf vermeintlich objektive biomedizinische Kriterien.

## Introduction

The emergence of technoscience is frequently understood as an epistemologically relevant trend toward increasingly close interactions of science and technology within processes of knowledge production as well as a shift of scientific interest toward the successful manipulation and “creation” of objects and artifacts. However, the formation of technoscience can also be seen as a socially and culturally significant phenomenon, in that the interrelated co-construction of scientific rationalities and technological devices has an enormous potential to shape and transform social practices, relations, and identities. One important field where this transformative potential of technoscience has been emphasized and vividly debated in recent years is medicine and health care (see for instance Clarke et al. 2003, 2009, 2010a; Burri and Dumit 2007; Gibbon and Novas 2008; Kollek and Lemke 2008; Mol 2008; Sulik 2009, 2011; Mathar 2010). In this article, I will therefore concentrate on the field of medicine and explore to what extent and with what consequences we can observe there what some authors have termed a process of “technoscientization” (Clarke et al. 2003, 2010b), that is, the transformation of medicine into a field a technoscientific practices.

Medicine appears to be a promising instance for an examination of the scope and limits of “technoscientization” since it has never simply been a field of “applied science” or “applied technology.” In spite of the undeniable scientization of medicine and the growing importance of medical technologies during the last decades, it has never ceased to be an experience-based social practice, a practice that is not only rooted in the professional experience of doctors but also in the personal and bodily illness experiences of the patients. Thus, it is an important question to ask whether these experiences and the related social identities are actually transformed into “technoscientific identities” as Clarke and co-authors argue (Clarke et al. 2003), that is, identities that are primarily based on scientific classifications (such as persons genetically “at risk”) and corresponding technological procedures (e.g., genetic testing, genome sequencing, or neuroimaging). Therefore, in order to probe the significance as well as the possible limits of the concept of technoscience as applied to the field of medicine, I will examine whether or not there is evidence of the more or less subtle inscription of technoscientific identities or “technoscientific illness identities” (Sulik 2009, 2011) on patients and their families, in particular on rare disease patients and their organizations.^1^ The article thus aims at investigating to what extent technoscientific illness identities are adopted or modified or rejected by these organizations in their discourses and practices. I consider this an illuminating empirical example since in social science research, patient associations are frequently linked with questions of identity formation.^2^ In addition, many rare disease patient organizations are on the one hand engaged in advocating and funding biomedical, frequently genetic, research into the causes and possible therapies of “their” diseases. One could presume, therefore, that this might make them receptive to the adoption of technoscientific definitions of their conditions. On the other hand, being self-help groups of affected persons or their parents, they are confronted with everyday experiences of illness that often require better health care and unspectacular symptomatic therapies which usually are beyond the interests of technoscientific biomedical and genetic research. Furthermore, fears of genetic discrimination or the rise of new eugenic practices have not entirely disappeared among rare disease patients and their relatives (Taussig et al. 2003; Lemke 2006). These facts might render patient organizations skeptical of or even resistant to biomedical knowledge claims. It comes as no surprise that such a focus on the illness identities of rare disease patients and their associations also touches on the highly influential concept of “biosociality,” introduced by Paul Rabinow (1996) during the 1990s. With this term, Rabinow refers to new kinds of social groups and communities which form around a certain biological feature such as a specific genetic variation. Against this background, biosociality plays a remarkably ambivalent and contested role in current discussions on biomedicine and technoscience: While for some authors, such as Clarke et al. 2003, biosociality is an essentially technoscientific concept promoting the adoption of technoscientific identities, for other scholars (e.g., Rose 2007), it rather seems to indicate that biomedical knowledges and practices can successfully be transformed and acculturated by “biosocial” groups such as patient associations.

In what follows, I would, first, like to very briefly sketch what might be understood by the terms “technoscientization” of medicine and “technoscientific identity” referring mainly to a widely received paper published in 2003 by the American sociologists Adele Clarke and colleagues. Then I will address the complex relationships between biomedicine and rare disease patient organizations as well as the ambivalent role the concept of biosociality plays in this context. Subsequently, drawing on preliminary results of current research at the University of Augsburg as well as on empirical work done by other authors, I will illustrate how rare disease patient associations deal with biomedical knowledge and whether or not they adopt technoscientific illness identities. Finally, I will draw some conclusions referring to the reach of the concept of technoscience in the field of medicine as well as to the ambiguities of the concept of biosociality.

## Biomedicine as technoscience: inscribing technoscientific illness identities?

In their already mentioned paper, Adele Clarke and co-authors understand the “technoscientization” of medicine as one of five key processes of what they phrase the “biomedicalization” of health and illness (Clarke et al. 2003).^3^ In general, they see this process of technoscientization as “part of major shifts in the social organization of biomedicine itself, the objects of biomedical knowledge production, the ways in which biomedicine intervenes, and the objectives with which it does so” (Clarke et al. 2003: 173). More precisely, technoscientization can be characterized by the fact that biomedical innovations are “increasingly likely to be hybrid ones that are generated simultaneously through sciences *and* technologies *and* new social forms” (ibid.—original emphasis). A more recent definition is quite similar; here, the authors speak of the “technoscientization of biomedical practices where interventions for treatment and enhancement are progressively more reliant on sciences and technologies, are conceived in those very terms, and are ever more promptly applied” (Clarke et al. 2010b: 2).^4^ Clarke et al. (2003: 173) describe technoscientization, more specifically, by three overlapping dynamics, first computerization and data banking, second molecularization and geneticization of biomedicine and drug design, and third, medical technology design, development and distribution, as for instance the design of new kinds of implants. The authors sum up that the ongoing technoscientization in these three areas “is at the heart of biomedicalization” (ibid.: 176).

Another one of the five basic processes of biomedicalization is characterized by Clarke et al. as the “transformation of bodies and identities,” and particularly as the construction of “new individual and collective technoscientific identities” (ibid.: 163).^5^ By technoscientific identities, the authors understand “the new genres of risk-based, genomics-based, epidemiology-based, and other technoscience-based identities” (ibid.: 182). Such identities are “produced through the application of sciences and technologies to our bodies directly and/or to our histories or bodily products including images” (ibid.). As the authors add, these new genres of identities, such as being carriers of genetic susceptibilities, are “frequently inscribed upon us, whether we like them or not” (ibid.). Again, Clarke and colleagues list a number of more specific ways that technoscientific biomedicine engages in processes of identity formation (ibid.: 182–183). The first one they mention is the use of technoscientific applications in order to attain a desired, yet previously unavailable social identity, for instance becoming a mother or father by means of reproductive technologies. Second, biomedicalization imposes new mandates and performances to be incorporated into one’s sense of self, for instance being proactive and prevention-conscious. The third trend implies the creation of new categories of health-related identities and the redefinition of old ones through the use of biomedical techniques such as genetic risk assessment. Thus, one’s identity can shift from “healthy” to “sick,” or from “low risk” to “high risk” and ostensibly being in need of specific biomedical prevention (Fosket 2004, 2010). Fourthly, biomedicalization also enables “the acquisition and performance of identities as patients and communities through new technoscientific modes of interaction, such as telemedicine” (ibid.: 183; see on this also Mathar 2010).

In a recent study, Gayle Sulik (2009) has developed the concept of a “technoscientific illness identity,” both drawing on the work of Clarke and coauthors and refining it. She describes a technoscientific illness identity as “a type of illness identity that involves applying biomedical information and characteristics to a persons’s sense of self (…). Rather than simply possessing a particular biomedical marker or classification, the person identifies so strongly with it that he or she integrates the classification into his or her identity.” (Sulik 2009: 1062).^6^ A person may, for instance, learn that she is genetically predisposed to a particular medical condition; in this situation, adopting a technoscientific illness identity means that this person begins “to think of herself as *pre*-*diseased*” (ibid.—original emphasis) and act accordingly. The person does not only *have* a biomedical classification but tends to *become* this classification (ibid.: 1060). Technoscientific illness identities thus are not merely inscribed upon patients but are, at least to some degree, accepted and actively adopted, if, probably, without fully realizing all of their implications (see also Clarke et al. 2003: 183).

Of course, some of the features of the technoscientization of biomedicine listed by Clarke et al. are debatable. Apart from the fact that the five key processes are introduced rather unsystematically, there is, for instance, at least occasionally a subtle and unacknowledged tendency toward a sort of technological determinism in their argument. Nevertheless, I would like to use their description of technoscientization as a heuristics, or as an “analytics” as the authors themselves suggest (Clarke et al. 2010b: 26), in order to explore and assess the actual impact of technoscientific biomedicine on rare disease patient associations’ practices and identities. There can be little doubt that the “successful” imposition of individual or collective technoscientific illness identities would be an important element of the “technoscientization” of contemporary societies. However, the scope and consequences of such transformations of identities remain open to empirical research: As Clarke and colleagues themselves emphasize, biomedicalization is not a “technoscientific tsunami that will obliterate prior practices and cultures” (Clarke et al. 2003: 184–185). They admit as well that the meanings of the emerging identities are negotiated in heterogeneous ways (idid. 182).^7^ Accordingly, in her qualitative study on women diagnosed with breast cancer, Sulik found out that a technoscientific illness identity has been adopted by no more than 27% of those women who used medical information in order to understand their situation (Sulik 2009: 1064).

## Biomedicine, rare disease patient organizations, and biosociality

Since the 1990s, many rare disease patient organizations which usually were founded as self-help groups of patients and their relatives have started to engage with scientific research into the causes and possible therapies of their respective diseases (see Epstein 2008). The resulting interrelations of these organizations and biomedicine are highly complex and not easy to describe. On the one hand, there is, of course, considerable overlapping of the interests and objectives of patient organizations and biomedicine. This holds true, first and foremost, with respect to biomedical research into the causes of disease and new therapeutical options. On the other hand, however, there might also be tensions and ambivalences regarding, for instance, the priorities of research, the understanding of disease, or the intricate problems of genetic testing or prenatal diagnosis. In this section, I will briefly elaborate, first, on these multifaceted interrelations of biomedical research and rare disease patient organizations and, second, draw attention to the question of whether and how the complexities of these interrelations are expressed in the concept of biosociality. I will argue that the above-mentioned contrasting interpretations of this concept originate, at least to some extent, from the fact that the ambivalent relationships between biomedicine and patient organizations are mirrored in ambiguities of the concept itself.

It is beyond any doubt that rare disease patient organizations have largely benefited and will continue to benefit from biomedical research, particularly from technoscientific genetic research (although not all rare diseases are associated with genetic variations). Discovering the genes and gene variants that are held to be responsible for a specific disease obviously can foster the scientific understanding of the pathogenic mechanisms and give rise to hopes for new and effective therapies (Novas 2007). The availability of a genetic test for the disease can put an end to the horrible odysseys, familiar to rare disease patients and their parents, from one doctor or clinic to the next one without receiving a reliable diagnosis. In addition, when biomedicine reveals the fact that rare diseases, such as neurodegenerative diseases or muscular dystrophies, are indeed *diseases* with biological causes, not erratic defects that breed strange kinds of beings, it supports patients in their struggles for social and medical recognition. Likewise, biomedicine can offer these patients new collective identities and create opportunities for political mobilization and active participation in research and research politics.

All these aspects have impressively been illustrated by Michel Callon and Vololona Rabeharisoa referring to the example of the *Association francaise contre les myopathies* (AFM), the French organization of muscular dystrophy (MD) patients which is able to fund biomedical research with comparatively large amounts of money: “In 1950 MD patients were excluded from common humanity; 30 years later they were considered as human beings in their own right. They had crossed the border, back into the realm of human beings, where they were simply *singularized* by a few genetic particularities. In the late 1950s they were undifferentiated MD patients; since the 1980s they have been unfortunate human beings affected by a particular disease, generally monogenetic, which explains why their bodies are so dramatically crippled and why they die earlier than other people. Uncertainties remain, especially on the possibility of preventing diseases and of finding therapies quickly. But many facts have been produced that make it possible to define options and to elaborate strategies. Professional networks—researchers, doctors, occupational therapists, and care officers—have been established, and patients with their families have formed groups to work together.” (Callon and Rabeharisoa 2008: 234–235—original emphasis).

As these authors summarize, MD patients have gone “from a situation of passive exclusion to one of active inclusion” (ibid.: 235), not least with the support of biomedical, genetic research. More specifically, the AFM in its overall strategy followed both a “path to cure” and a “path to citizenship”; on the path to cure, it adopted a “genetic identity” which helped it to engage in biomedical research and which finally could be transformed into a “genetic citizenship” (idid.: 236). In another paper, the authors even ascribe a “civilizing” effect to “the gene” (understood as an “actant” in the actor-network-theory terminology) (Callon and Rabeharisoa 2003: 201): “By showing that a defect is in fact a small genetic accident, the AFM demonstrates that we are all just one or two genes away from being MD patients. Genes are not content just to make particular and general interests compatible; they also produce solidarity and compassion.” (Ibid.: 200).^8^

Another example of a patient association that successfully collaborates with biomedical research and even became a research organization itself is *PXE International*, an advocacy organization which initiates, funds, and conducts research on *pseudoxanthoma elasticum* (PXE), a rare genetic disorder that causes vision, skin, and arterial defects (Terry et al. 2007; see also Novas 2007). PXE International did not only fund biomedical research that, in 2000, was successful in identifying the genetic mutations associated with PXE; in addition, the founders of the organization, Sharon and Patrick Terry, themselves participated in this research and therefore are named as co-inventors on the patent for the discovered gene. They assigned their rights to the foundation, as did the scientists who had been involved in identifying the gene (Terry et al. 2007: 161). In addition, PXE International founded a blood and tissue bank, established a genetic test for the disease, funded the development of an animal model, conducted clinical trials, and mentored other rare disease advocacy groups (Terry et al. 2007).

Yet, despite these successful examples, there might also be a number of tensions between biomedical research and rare disease patient organizations. Two of these tensions are particularly important: First, genetic research up to now is far more effective in identifying genes “causing” diseases and in developing diagnostic tests than in finding new, promising therapies. As Aaron Panofsky has pointedly put it in a recent paper, “Research supported by rare genetic disease PAOs [patient advocacy organizations] has almost never achieved its ostensible goal of developing cures or even effective treatments.” (Panofsky 2011: 34)^9^ This holds, for instance, for the case of cystic fibrosis (CF): While the gene associated with the disease has been discovered as early as 1989, there is still no cure available. Stockdale and Terry (2002) have retrospectively criticized the US patient organization Cystic Fibrosis Foundation (CFF) for its “narrow focus on ‘curing’ CF” by means of gene therapies and for directing extensive resources almost exclusively to basic research during the 1990s. At the same time, they argue, “other issues, such as ready access to affordable medications and care, receive less attention than they might warrant” (Stockdale and Terry 2002: 82). They add that this neglect applies to the difficult problems of genetic testing as well that had been raised by the discovery of the gene.^10^ In general, technoscientific biomedicine appears to be interested primarily in basic research, while the aim of patient organizations clearly lies in what Terry et al. (2007: 158) have termed “health outcomes” and “immediate translational benefits.” These authors therefore argue that “research that has long-term benefits for understanding general disease mechanisms must be balanced with research that has more immediate applications” (ibid.: 163; see also Stockdale and Terry 2002: 80). At any rate, this is a potential source of tension or even conflict of interests between biomedical research and rare disease patient organizations, particularly when we take into account that patient organizations frequently lack appropriate means to control what kind of research the scientists whom they fund actually do.

Second, the gap between diagnostics (frequently by means of genetic testing) on the one hand and effective therapies on the other raises a number of new problems and difficult questions which are controversially debated within and among different patient organizations. These problems include the creation of new categories of people situated between health and illness, such as carriers of so-called susceptibility genes with heightened disease risks or carriers of disease genes, so-called heterozygotes, for instance healthy siblings of children affected by autosomal recessive diseases. These persons who themselves are healthy but potentially carry the deleterious gene might be confronted with calls for premarital testing or prenatal testing of their children. Thus, the mismatch of diagnostics and therapies almost inevitably moves the issue of prevention by means of genetic testing and screening to the foreground. While some patient organizations, as for instance the Beyond Batten Disease Foundation in the US, are advocating large-scale genetic screening for rare diseases,^11^ others such as “Little People of America” (LPA), a self-help organization of people with achondroplasia, a form of heritable, single-gene dwarfism, are more reluctant to prenatal genetic testing fearing the proliferation of eugenic practices (Taussig et al. 2003). Still other organizations appear to be internally split with regard to these questions. Thus, at least some patient organizations do not conform with technoscientific practices of reconfiguring social identities which, for the time being, imply a tacit shift from therapy to prevention.

How do these complex interrelations of biomedicine and patient organizations become manifest in contradictory interpretations of the concept of biosociality—and in what way do these interpretations mirror the ambiguities of the concept itself? Remarkably, Clarke and her co-authors understand biosociality as “the major framing of technoscientific identities” (Clarke et al. 2003: 183), as a new mode of social relations “deeply linked” to living with such identities (Clarke et al. 2009: 23). With regard to disability activists, Bill Hughes similarly criticizes those groups who understand themselves as biological citizens or biosocial groups for embracing a “medicalised concept of self-identity” as well as for sharing a questionable “optimism about the benefits of technology and medical science” (Hughes 2009: 686, 680). As a result, he observes the bifurcation of disability activism into one camp that is advocating a social model of disability and another (“biosocial”) camp that is adopting biomedical models of disability and disease (ibid.: 685). Such critical understandings of biosociality seem to be not entirely unjustified, for Rabinow himself has introduced the concept with a strong reference to communities and identities which arise out of a biomedical classification and refer to a specific genetic condition, for instance “groups formed around the chromosome 17, locus 16,256, site 654,376 allele variant with a guanine substitution” (Rabinow 1996: 102). Nevertheless, the characterization of biosociality as deeply linked to questionable technoscientific identities contrasts with a widespread view which understands it primarily as the basis for creating new forms of collective action, as an empowerment of patients and/or their parents, especially with regard to their interactions with biomedicine. Rose (2007: 144–147), for instance, conceives of biosociality in terms of making claims for “active biological citizenship” and of lay actors’ participation in the cooperative production of medical knowledge. In particular, patient organizations are credited in recent social science research with the ability not only to contribute to the creation of novel forms of knowledge and social relations but also to escape the imposition of technoscientific identities.

These diverging interpretations point to an underlying ambiguity of the concept itself, for it leaves open whether biosocial groups are held to simply arise out of supposedly *objective* biological or genetic conditions (“the chromosome 17, locus 16,256, site 654,376 allele variant with a guanine substitution”) with which they identify and to which they try to adjust their lives. Such a reading is at least partly supported by Rabinow himself when he writes that biosocial groups “will have medical specialists, laboratories, narratives, traditions, and a heavy panoply of pastoral keepers to help them experience, share, intervene, and ‘understand’ their fate” (Rabinow 1996: 102). Or do these groups, quite to the contrary, constitute themselves by re-defining the ostensibly given biological basis of their identities thus creating truly bio*social* identities and possibly questioning biomedical knowledge claims? This interpretation, too, can draw upon Rabinow’s considerations, for instance when he contrasts biosociality with (“biologistic”) sociobiology: “If sociobiology is culture constructed on the basis of a metaphor of nature, then in biosociality nature will be modeled on culture understood as practice.” (Ibid.: 99). Thus, while the concept of biosociality itself might, in principle, be open to both interpretations, in recent social science discussions, it has frequently been narrowed to the idea of fixed biological identities which are defined by biomedicine and held to form a stable basis on which biosocial groups (e.g., patient organizations) operate.^12^ Understood in this way, biosociality is indeed a “framing of technoscientific identities”; however, in the following sections, I will explore whether or not there are possibilities to conceive of the concept in different, less deterministic ways.

## Rare disease patient associations and technoscientific illness identities

How do rare disease patient organizations deal with biomedical knowledge claims and technoscientific illness identities? In this section, I will focus on two aspects which appear to be illuminating with regard to these questions: first, the issues of research priorities and research funding and second, those of illness experiences and identities of the patient organizations and/or their members.

One important impact that biomedicine doubtlessly has on rare disease patient associations, at least on those concerned with supposedly genetic diseases, is what Clarke et al. (2003: 182) have termed the “creation of new categories of health-related identities and the redefinition of old ones.” Since the 1990s, in the context of Human Genome Research, for many rare diseases, the genes or the gene variants held to be causally responsible have been identified. As a consequence, these diseases were no longer conceived as incurable ones but redefined, according to the promises of biomedicine, as potentially or even probably curable ones by genetic or some other sort of therapy (Novas 2007: 11). This shift is anything but trivial, since it constructs or “interpellates,” as Langstrup (2011) puts it referring to the French philosopher Louis Althusser, rare disease patients (and their organizations) as future users of potentially new biomedical therapies and thus transforms both their social and illness identities. Many researchers as well as representatives of patient groups believed after the discovery of “the gene for” their illness that therapy seemed to be “just around the corner” (see Stockdale 1999). Nevertheless, this shift from incurable to supposedly curable diseases has been utterly important for rare disease patient organizations since it offered them opportunities for meaningful participation in biomedical research with the aim to find therapies for their respective diseases. In reaction to the promise of novel, effective cures, many patient associations started to support or intensified their support for biomedical research concentrating primarily on basic research.

However, although there are various reasons to consider this development a positive achievement, it still remains an open question to what extent rare disease patient organizations do (and should) subscribe to the objectives, research agendas, and interests of technoscientific biomedicine. As already mentioned, some scholars have warned patient organizations against too readily buying into the “popular dream of breakthrough medicine” (Wailoo and Pemberton 2006: 168; see also Stockdale and Terry 2002) because this might distract their attention from other important tasks concerning symptomatic therapies and health care.^13^ To put it differently, is it sufficient for rare disease patient organizations simply to focus on the *acceleration* of science, as Carlos Novas (2007: 17) argues, or should they also be concerned with *realigning* medical research toward paying more attention to scientifically perhaps unattractive, yet therapeutically valuable short- or middle-term benefits for those who are currently affected by the disease?

As our findings in a current research project suggest,^14^ the impact that the “gene hype” of the 1990s had at least on some patient associations subsequently has been replaced by a more nuanced and skeptical view. As one of our interview partners from a German patient organization has put it, “The idea, that I correct the defective gene and everything is fine, is definitely over.” Instead, research that is funded by rare disease patient associations today is directed to gradual improvements of symptomatic therapies and health-care practices no less than to basic genetic research. Thus, with regard to research priorities and more general attitudes toward the diseases, the strong influence that technoscientific biomedicine exerted on some rare disease patient groups during the 1990s and early 2000s appears to be a temporal phenomenon rather than a stable long-term trend (see on this also Sect. 5). However, the extent to which rare disease patient organizations refrain from the promises of genetic medicine seems to depend on the characteristics of the respective disease as well as on the organizational structure of the groups. As to the first aspect, it is clear that the vision of “repairing” the faulty gene is more compelling when the disease takes a rapid and unstoppable lethal course than when the patients still have a life expectancy of 20 or more years after onset of the disease. As regards the organizational structures, it seems that those associations that understand themselves and act mainly as self-help groups or are closely tied to self-help groups are more reluctant to adopt biomedical views and promises than organizations (e.g., foundations) whose aim is primarily or even exclusively to fund and promote medical research.

Nevertheless, beyond the question of research funding and research priorities, *molecularization* and *geneticization*, which have been identified by Clarke and colleagues as one of three key elements of the technoscientization of medicine, doubtlessly exert considerable influence on the understanding of disease in patient associations. For instance, classifications of the respective diseases and of the patients along diverse variants of the “gene defect” are quite usual. Yet the practical impact on patients’ illness identities of such biomedical knowledge claims often seems to be rather limited or is even limiting itself by producing paradoxical effects. In this respect, I would like to emphasize two aspects: First, the members of patient organizations frequently observe that there are only weak and uncertain links between genotypes and phenotypes. One of our interview partners, the founder of a very small German patient association, gave the example of a family having two children, a boy and a girl, with exactly the same genetic variants associated with the disease. But while the boy died at the age of 10, his sister, though affected by the disease, was still alive and comparatively well. Thus, even when rare disease patient associations and their members adopt a “genetic” illness identity, it often is a phenotypically differentiated identity rather than a purely genotypical one.^15^ To put it differently, although the patients *have* a genetic classification, they do not necessarily *become* this classification (Sulik 2009), since the lived experience of illness does not always correspond to such genotype-related classifications.

The second aspect that is worth mentioning in this context is important with regard to the concept of biosociality as well: Even conditions by which only very few persons are affected often differentiate into several genetic variations with specific therapies being required for each of these subtypes. As an interview partner mentioned, this may result in difficult constellations within the patient organizations when, for instance, there appear to be better chances to develop therapies for a specific subtype than for others. Thus, biomedical research that continues to fragment the genetic basis of diseases into various subtypes is likely to confront the patient associations with problems of identity, cohesion, and solidarity. At the same time, it tends to undermine the idea that technoscientific knowledge would produce an objective and stable basis for building new forms of biosocial identities. As it seems, many patient groups, again, react to such challenges by adhering to more phenotype-based conceptions of the disease. In addition, solidarity among the group members frequently is built on the common *social* experience of having been neglected by the mainstream of medical research as well as on the joint efforts to change this situation rather than on shared biological traits (see also Huyard 2009b).^16^ This might be one of the main reasons why umbrella organizations such as the German ACHSE (*Allianz Chronischer Seltener Erkrankungen*) covering a broad range of heterogeneous rare diseases appear to be quite successful in promoting among its member organizations a shared social and political identity of “being rare.”

## Conclusions: the limits to technoscientization

Apparently, the interrelations of biomedicine, technoscience, and rare disease patient organizations are too complex and heterogeneous that one could reasonably speak of an unambiguous trend toward the “technoscientization” of social identities and practices in this medical field.^17^ Although the impact of biomedicine on rare disease patient associations should by no means be underestimated, the formation of technoscientific illness identities in the strict meaning suggested by Sulik (2009) seems to be not very common among these organizations’ representatives or members. While they do, of course, accept and refer to biomedical classifications of their diseases, they rarely identify so strongly with these classifications that they would integrate them into their own identities or those of their affected children. One of the main reasons for this reservation can be seen in the fact that biomedical and genotypical classifications frequently do not match the lived experiences of illnesses and their (“phenotypical”) peculiarities and uncertainties. In addition, since genetics-based research to date has largely failed to provide effective therapies, the attention of many rare disease patient organizations has shifted from the promises of future “breakthrough cures” (back) to more immediate “health outcomes,” as Terry et al. (2007) have put it. Quite similarly, Henriette Langstrup (2011) has shown in her study on associations of patients suffering from more widespread diseases, such as diabetes, multiple sclerosis, Parkinson’s, or Alzheimer’s, that only few of these organizations responded positively to being “interpellated” as future users of stem cell technologies and therapies. Other associations, by contrast, considered it irresponsible to raise among their members unrealistic hopes for cure and therefore rejected the suggested identity of future users of stem cell technologies and did not support this strand of research (Langstrup 2011: 585f.).

As regards the contested concept of biosociality, it would be overstated to maintain that it is technoscientific in itself. It is, however, an ambiguous concept which leaves room for contradictory interpretations, as shown in Sect. 3. In any case, the formation of biosocial groups and identities is not so straightforward as Rabinow’s initial account might suggest, according to which biosocial collectives and identities simply emerge around a shared biomedical classification and genetic condition. Instead, it is subject to negotiation and contestation what the “biological” in biosociality actually means (see Wehling 2011); the same applies, of course, to the “social” which does not represent a fix and taken-for-granted identity either. Biosociality should therefore be conceived in the first place as a social context in which conceptions of health, disease and biology as well as social and illness identities are negotiated, both influenced by biomedicine and possibly resistant to it.^18^

In a recent reflection on the (short) history of the concept of biosociality, Rabinow himself pointed to the fact that both the concept and the hopes directed to technoscientific biomedicine, in particular to the mapping of the human genome, were influenced by the enthusiastic climate of the 1990s which he retrospectively termed the “Golden Age of Molecular Biosociality”: “There was hope, there was progress, there was a reason to be urgent even strident—there were reasons to want to be biosocial.” (Rabinow 2008: 190) According to Rabinow, that decade has been a formative period “when technology-driven advances in understanding were undeniable—a moment when it still seemed legitimate to have hope for dramatic, even definitive, diagnostic and therapeutic triumphs” (ibid.). Obviously, such hopes pinned on biomedicine and biosociality have at best partly been realized. As Rabinow admits, “the hopes and hype of the genomic decade have failed to provide adequate diagnostic or risk assessment tools or treatments based on them” (ibid.: 192). Thus, he adds that some of the limits of the concept of biosociality can now be seen “with more clarity”; it should therefore be understood as a heuristic tool rather than as “an epochal designation meant to characterize an age or era” (ibid.: 191). Hence, it might now be the right moment to analytically separate this concept from the historical context in which it has been coined and by which it has been shaped and to rethink it in order to investigate the complex and ambiguous relationships of biomedicine, illness identities, and patient associations under changing circumstances.

## Footnotes

1 “Rare disease” is merely a statistical classification (see Huyard 2009a on the history of the concept of “rare diseases”). Within the EU, a rare disease is defined as one by which no more than 5 in 10,000 persons are affected. In principle, these diseases have little or even nothing in common with respect to etiology, symptoms, or therapies, although many of them, yet not all, have a genetic origin, appear during childhood, and take a lethal course. However, the term “rare disease” should not hide the fact that, with an estimated number of 5,000–7,000 rare diseases, about 30 million people are affected only in the EU.

2 See for instance Callon and Rabeharisoa (2003), Novas (2007), and Langstrup (2011). The interrelations of genetic medicine and emerging new identities are investigated more generally in Atkinson et al. (2007).

3 See for more recent accounts Clarke et al. 2009 and Clarke et al. 2010b. The 2003 paper has been reprinted as a chapter in Clarke et al. 2010a. By the term “biomedicalization,” these authors understand a new, intensified, and technoscientifically transformed shape of medicalization (Clarke et al. 2003: 162). Medicalization, in turn, is usually conceived of as a process by which previously non-medical phenomena are defined in medical terms and treated with a medical intervention (see Conrad 2007: 5).

4 Basically, the authors draw on Bruno Latour’s conception of technoscience and emphasize that “science and technology are not easily distinguishable (…) but should instead be understood as co-constituted and hybrid” (Clarke et al. 2010b: 42, n. 1).

5 The other three basic processes of biomedicalization which I do not explicitly deal with here are, first, major political economic shifts resulting in a new biopolitical economy of medicine, health, illness, living, and dying; second, a new focus on health (in addition to illness and disease) as well as on optimization and enhancement and the development of risk and surveillance biomedicines; and third, transformations of the production, distribution, and consumption of biomedical knowledges (cf. Clarke et al. 2003: 166; Clarke et al. 2010b: 1–2). It becomes clear that there is much overlapping of the five basic processes, and the demarcations between them are not always clear-cut, but I cannot elaborate on this here (see for a partly different account of the current transformations of medicine Wehling and Viehöver 2011).

6 The concept of an “illness identity” has been developed by Kristin Barker (2002) using the example of fibromyalgia syndrome (FMS) patients. According to Barker (2002: 281–84), an illness identity is formed when patients situate their personal experiences onto public illness narratives and simultaneously use these narratives to understand themselves and their situations.

7 Therefore, at the end of their 2003 paper, they call for case studies “that attend to the heterogeneities of biomedicalization practices and effects in different lived situations” (Clarke et al. 2003: 185).

8 This statement is, however, not unproblematic since in other contexts, “the gene” may have adverse effects, for instance when individuals are stigmatized as “gene carriers” and exposed to the risk of genetic discrimination (Lemke 2006). Apparently, these more critical, potentially excluding aspects of “genetic identities” threaten to be underestimated as long as genetics research promises to provide cures for hitherto incurable diseases.

9 For Panofsky, it is therefore a “fascinating question” why patient organizations are interested in genetic research at all, “since diagnostic rather than therapeutic tools are the most likely near-term outcomes of such research” (Panofsky 2011: 34; see also Langstrup 2011).

10 For a more detailed account of the search for gene therapy for CF and its shortcomings, see Wailoo and Pemberton (2006: 61–115).

11 Batten disease is a fatal neurodegenerative disease that appears during childhood. The Beyond Batten Disease Foundation currently is supporting research to develop a single, easy, and inexpensive blood test to detect the gene mutations for hundreds of rare diseases, particularly autosomal recessive diseases such as Batten disease (http://www.beyondbatten.org/about-prevention.html, last access 10.9.2011).

12 See for more detailed criticisms of this narrow understanding of biosociality Lemke and Wehling 2009; Lemke 2010; Wehling 2011.

13 As Keith Wailoo and Stephen Pemberton argue, the “genetics revolution” up to now has “most transformed society in the areas of diagnosis and prevention. Therapy continues to be a vexing challenge. If the promise of breakthrough cures has often been unrealized, a large part of the problem is rooted in the stubborn asymmetries between scientific and technological models of disease and how bodies actually work.” (Wailoo and Pemberton 2006: 168).

14 The research project “Participatory Governance of Science” is conducted at the University of Augsburg and funded by the German Federal Ministry of Education and Research (BMBF) from 2009 to 2012. It focuses on the role of civil society organizations such as environmental and consumer organizations, health movements, or patient associations in the governance of science and technology. In this context, we conducted interviews with leading representatives of several German rare disease patient associations ranging from “classical” self-help groups to research-oriented foundations. Since the project is not yet completed, the findings referred to in this article are necessarily preliminary ones.

15 See for similar findings with regard to haematochromatosis the study by Bharadwaj et al. 2007. In addition, testing for genotypes can itself generate new uncertainties, as is the case with Huntington’s disease where genetic testing creates so-called “intermediate” persons who may or may not develop the disease (cf. Lemke 2004: 33–35).

16 Similarly, based on his study on rare disease patient advocacy organizations, Panofsky (2011: 39) is questioning the assumption that “the active assertion of a positive disease identity” is a strong part of these organizations’ strategies of promoting research.

17 This result does, however, not preclude the possibility of “successful” technoscientization in other medical areas. Mathar (2010), for instance, investigates the technoscientization of health care by telemedicine and telemonitoring of patients but points to the limits to such efforts as well.

18 The term “resistant biosociality” has been coined by Taussig et al. (2003: 66) with a view to the opposition to prenatal genetic testing for “dwarfism” of “Little People of America”, a rare disease patient association. This opposition has been driven mainly by fear of the emergence of eugenic practices.
